# Guidelines for administering gadolinium-based contrast agents to patients with renal dysfunction (version 3: revised may 20th, 2024)

**DOI:** 10.1007/s11604-024-01719-9

**Published:** 2025-02-12

**Authors:** Joint Committee of NSF and Use of Gadolinium Based Contrast Agents (Japan Radiological Society, Japanese Society of Nephrology)

**Affiliations:** Tokyo, Japan

The Joint Committee for NSF and Use of Gadolinium-Based Contrast Agents (GBCAs), a joint committee of the Japan Radiological Society and the Japanese Society of Nephrology, first announced guidelines for the use of GBCAs on July 25th, 2008 and revised them on September 2nd, 2009.

Nephrogenic systemic fibrosis (NSF) is a disease characterized by swelling, hardening, and pain of the skin after exposure to GBCAs. Disease progression can lead to limb contractures, severely limiting physical activity. There is currently no known cure, and there have been reports of death due to complications from NSF. Taking into consideration new knowledge acquired since the previous revision, the Joint Committee has revised the guidelines as follows:

1. Regardless of renal function, GBCAs should only be used when absolutely necessary for diagnosis. When administered, carefully follow indications and dosage stated in the respective drug’s package insert. GBCAs should never be used as a contrast agent for X-ray examinations such as CT or angiography.
2. Before performing a contrast enhanced MRI, evaluation of renal function (estimated glomerular filtration rate: eGFR) is recommended, except in emergency situations.
3. Conditions in which avoidance of GBCAs and substitution with another diagnostic modality is preferable are the following:
4. end-stage kidney disease in long term dialysis patients;
5. chronic kidney disease with eGFR less than 30 ml/min/1.73 m^2^ in non-dialysis patients;
6. acute kidney injury.

When substitution with another diagnostic modality is difficult, GBCA-enhanced MRI may be performed very carefully by considering the risks of NSF and following appropriate dosage of GBCA. If repeat examinations are necessary, perform them as far apart as possible.

*ESUR guidelines state that ideally, there should be seven or more days between two GBCA injections [1, 2].

[Reasons for revision].

GBCAs are designed to be administered safely by chelating the heavy metal gadolinium. When excretion of gadolinium is delayed due to reduced kidney function, the chelate is released and the toxicity of gadolinium becomes apparent. There is a large variation in chelate stability among GBCAs. Those associated with many cases of NSF are considered to have lower chelate stability [1, 3]. In Japan, these agents are no longer available (see Table 1).

**Table 1:** GBCAs sold in Japan (including those that are no longer commercially available)

Contrast agent	Abbreviation	Trade name	Comments
Gadoteridol	Gd-HP-DO3A	Prohance	
Gadoterate	Gd-DOTA	Magnescope	
Gadobutrol	Gd-BT-DO3A	Gadovist	
Gadoxetate sodium	Gd-EOB-DTPA	EOB, Primovist	Liver-specific contrast agent
Gadodiamide	Gd-DTPA-BMA	Omniscan	Currently unavailable
Gadopentetate	Gd-DTPA	Magnevist	Currently unavailable

The mechanism of NSF is not entirely clear. However, based on the most recent evidence for risks of developing NSF after exposure to GBCA [see (a) to (f) below], even patients with reduced renal function or patients on dialysis have very low risk in developing NSF, as long as GBCAs currently available in Japan are used. The disadvantages to the patient for being denied GBCA-enhanced MRI should also be taken into consideration and minimized. The previous guidelines stated that patients with “end stage renal disease in long-term dialysis patients, chronic renal failure with eGFR less than 30 ml/min/1.73 m^2^, and acute renal failure” should not, as a general rule, receive GBCAs, and that in these patients “GBCA-enhanced MRI should be replaced with an alternative examination.” Under the current revision, this has been revised to “In these patients, avoidance of GBCAs and substitution with another diagnostic modality is preferable.” in addition to “when substitution with another diagnostic modality is difficult, GBCA-enhanced MRI may be performed very carefully by considering the risks of NSF and following appropriate dosage of GBCA,” and “if repeat examinations are necessary, perform them as far apart as possible.”

- Woolen et al. performed a meta-analysis on 16 references and found that there were no cases of NSF for group II GBCAs (stable chelates with minimal risk of NSF) even in patients with CKD stage 4 or 5 (including dialysis patients), with a 95% CI of 0.07% [4]. This meta-analysis included a report [5] from Japan on Gadobutrol administered to 3337 patients. Among these patients, there were no reports of NSF although only five patients were at CKD stage 4 or 5.
- Amet et al. reported that there were no cases of NSF among 287 dialysis patients who received mostly GBCAs with high stability [6]
- Alfano et al. reported that there were no cases of NSF among 344 dialysis patients who underwent 551 examinations using a GBCA with high stability (Gadoterate) [7].
- Michaely et al. did a prospective study on a total of 908 patients including 83 dialysis patients, 201 non-dialysis patients with CKD stage 4 or 5, and 586 patients with CKD stage 3, and stated that there were no cases of NSF [8].
- Starekova et al. reported on 7820 examinations on 5351 patients who were administered the liver-specific agent Gadoxetic sodium [9]. There were no cases of NSF. The study included 133 patients with CKD stage 4 or 5, and 27 dialysis patients.
- Attari et al. performed a systematic review on 639 patients with biopsy-confirmed NSF, and found that only one case occurred after administration of a highly stable GBCA [10].

## Appendix

The following are widely used international guidelines. Please refer to original references for details.

- 1. ESUR guidelines on contrast agents, version 10.0. (European Society of Urogenital Radiology) [1] These guidelines state that patients with eGFR less than 15 ml/min/1.73 m^2^ and dialysis patients are at risk of NSF. They also state that GBCAs with lowest risk of NSF (Gadoteridol, Gadoterate, Gadobutrol) should be used for patients with eGFR less than 30 ml/min/1.73 m^2^ with caution, and that there should be at least seven days between two injections.
- 2. ACR manual on contrast media, ver. 2023. (ACR Committee on Drugs and Contrast Media) [3] These guidelines state that for dialysis patients, contrast-enhanced CT is a better choice than contrast-enhanced MRI, and for patients with eGFR less than 30 ml/min/1.73 m^2^, group II GBCAs (Gadoteridol, Gadoterate, Gadobutrol) should be used. There are no reports of NSF for group III GBCAs (Gadoxetate sodium), but these guidelines state that data are insufficient.
